# Synthesis and crystal structure of poly[ethanol(μ-4-methyl­pyridine *N*-oxide)di-μ-thio­cyanato-cobalt(II)]

**DOI:** 10.1107/S2056989024009058

**Published:** 2024-09-20

**Authors:** Christian Näther, Inke Jess

**Affiliations:** a Institut für Anorganische Chemie, Universität Kiel, Germany; University of Kentucky, USA

**Keywords:** synthesis, crystal structure, cobalt thio­cyanate, coordination polymer, layered network, 4-methyl­pyridine *N*-oxide

## Abstract

Reaction of 4-methyl­pyridine *N*-oxide and Co(NCS)_2_ in ethanol as solvent accidentally leads to the formation of single crystals of Co(NCS)_2_(4-methyl­pyridine *N*-oxide)(ethanol) or [Co(NCS)_2_(C_6_H_7_NO)(C_2_H_6_O)]_*n*_. The asymmetric unit of the title compound consists of one Co^II^ cation, two crystallographically independent thio­cyanate anions, one 4-methyl­pyridine *N*-oxide coligand and one ethanol mol­ecule on general positions. The cobalt cations are sixfold coordinated by one terminal and two bridging thio­cyanate anions, two bridging 4-methyl­pyridine *N*-oxide coligands and one ethanol mol­ecule, with a slightly distorted octa­hedral geometry. The cobalt cations are linked by single μ-1,3(*N*,*S*)-bridging thio­cyanate anions into corrugated chains, that are further connected into layers by pairs of μ-1,1(*O*,*O*)-bridging 4-methyl­pyridine *N*-oxide coligands. The layers are parallel to the *bc* plane and are separated by the methyl groups of the 4-methyl­pyridine *N*-oxide coligands. Within the layers, intra­layer hydrogen bonding is observed.

## Abstract 2

In the crystal structure of the title compound, the Co^II^ cations are octa­hedrally coordinated by two bridging and one terminal thio­cyanate anions, two bridging 4-methyl­pyridine *N*-oxide coligands and one ethanol mol­ecule and linked into chains by single μ-1,3-bridging anionic ligands that are further connected into layers by pairs of μ-1,1(*O*,*O*)-bridging 4-methyl­pyridine *N*-oxide coligands.

## 1. Chemical context

Coordination polymers based on transition-metal thio­cyanate coordination polymers are characterized by a pronounced structural variability, which can partly be traced back to the variety of coordination modes of this anionic ligand. This includes mainly the terminal coordination, the μ-1,3(*N*,*S*) and the μ-1,3,3(*N*,*S*,*S*) bridging modes. With μ-1,3(*N*,*S*) bridging anionic ligands and octa­hedrally coordinated metal cations the majority of compounds consist of *M*(NCS)_2_ chains, in which the metal centers are connected by pairs of thio­cyanate anions. In most cases an all-*trans* coordination is found, which leads to the formation of linear chains (Rams *et al.*, 2017*a* ▸, 2020 ▸; Mautner *et al.*, 2018*a* ▸,*b* ▸). Linear chains are also observed if the co-ligands are in *trans*-positions and the thio­cyanate N and S atoms are in *cis*-positions (Rams *et al.*, 2017*b* ▸). For the other or *cis*–*cis*–*trans* and the all-*cis* coordination, corrugated chains are observed (Maji *et al.*, 2001 ▸; Marsh, 2009 ▸; Shi *et al.*, 2006*a* ▸, 2007*a* ▸; Böhme *et al.*, 2020 ▸). In contrast, chain compounds in which the metal cations are linked by single μ-1,3-bridging thio­cyanate anions are rarer (Palion-Gazda *et al.*, 2015 ▸; Neumann *et al.*, 2018 ▸).

We have been inter­ested in transition-metal thio­cyanates for a long time, with special focus on Co(NCS)_2_ chain compounds with pyridine derivatives as coligands (Rams *et al.*, 2017*a* ▸,*b* ▸, 2020 ▸; Böhme *et al.*, 2022 ▸). Later we also used pyridine *N*-oxide derivatives as coligands, because they can additionally connect metal cations *via* the μ-1,1(*O*,*O*) bridging mode. In this regard we became inter­ested in 4-methyl­pyridine *N*-oxide as coligand. With this ligand, two compounds with the composition Co(NCS)_2_(4-methyl­pyridine-*N*-oxide) (Zhang *et al.*, 2006*a* ▸) and Co(NCS)_2_(4-methyl­pyridine *N*-oxide)(meth­anol) (Shi *et al.*, 2006*a* ▸) were reported in the literature. In the first compound, the cobalt cations are octa­hedrally coordinated by two N- and two S-bonding thio­cyanate anions and two bridging 4-methyl­pyridine *N*-oxide coligands and are connected by pairs of bridging anionic ligands into corrugated chains, which are further connected into layers by the 4-methyl­pyridine *N*-oxide coligands. In the second compound, the cobalt cations are octa­hedrally coordinated by one terminal and two bridging thio­cyanate anions, two bridging 4-methyl­pyridine *N*-oxide coligands and one methanol mol­ecule. The metal cations are linked by alternating pairs of μ-1,3-bridging thio­cyanate anions and μ-1,1(*O*,*O*) bridging 4-methyl­pyridine *N*-oxide coligands into chains. In the course of our investigations we have synthesized two discrete complexes with the composition Co(NCS)_2_(4-methyl­pyridine *N*-oxide)_3_ and Co(NCS)_2_(4-methyl­pyridine *N*-oxide)_4_ that show a trigonal–bipyramidal or an octa­hedral coordination (Näther & Jess, 2024*a* ▸). The first complex can easily be synthesized from methanol, but in some of these batches an additional 4-methyl­pyridine *N*-oxide compound was detected. Later we have found that a compound with the composition Co_2_(NCS)_4_(4-methyl­pyridine *N*-oxide)_4_(methanol)_2_ was obtained as a by-phase, in which the Co^II^ cations are linked by pairs of μ-1,1-bridging 4-methyl­pyridine *N*-oxide coligands into centrosymmetric dinuclear units (Näther & Jess, 2024*b* ▸).

Some of the 4-pyridine *N*-oxide compounds mentioned above can also be prepared in ethanol as solvent. However, in some of these batches traces of an additional product were detected by X-ray powder diffraction and therefore a large number of crystallization experiments were performed. In one of these batches crystals suitable for single-crystal X-ray diffraction were accidentally obtained, which proved that a compound with the composition Co(NCS)_2_(4-methyl­pyridine *N*-oxide)(ethanol) had formed.
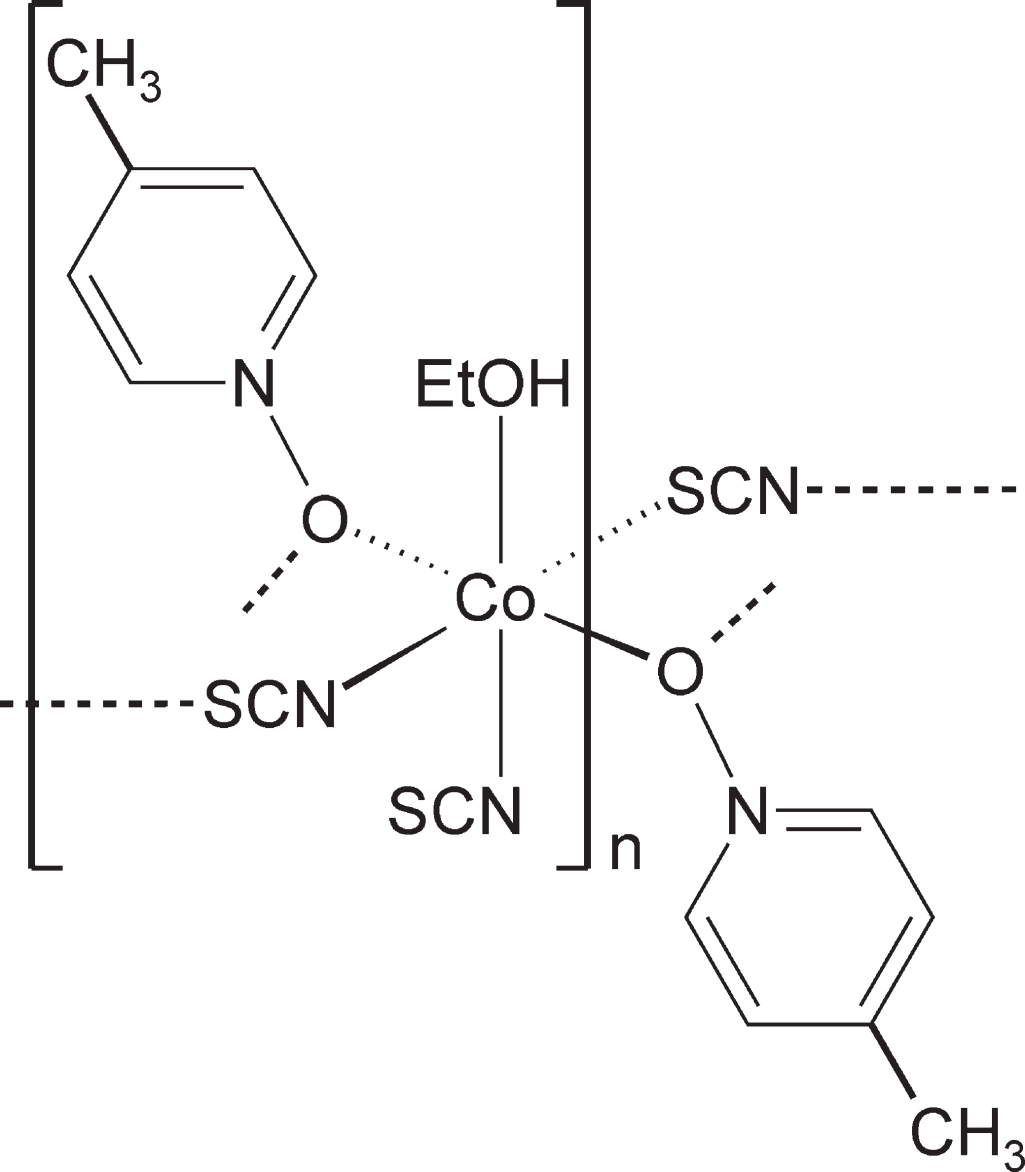


## 2. Structural commentary

**Figure 1:**
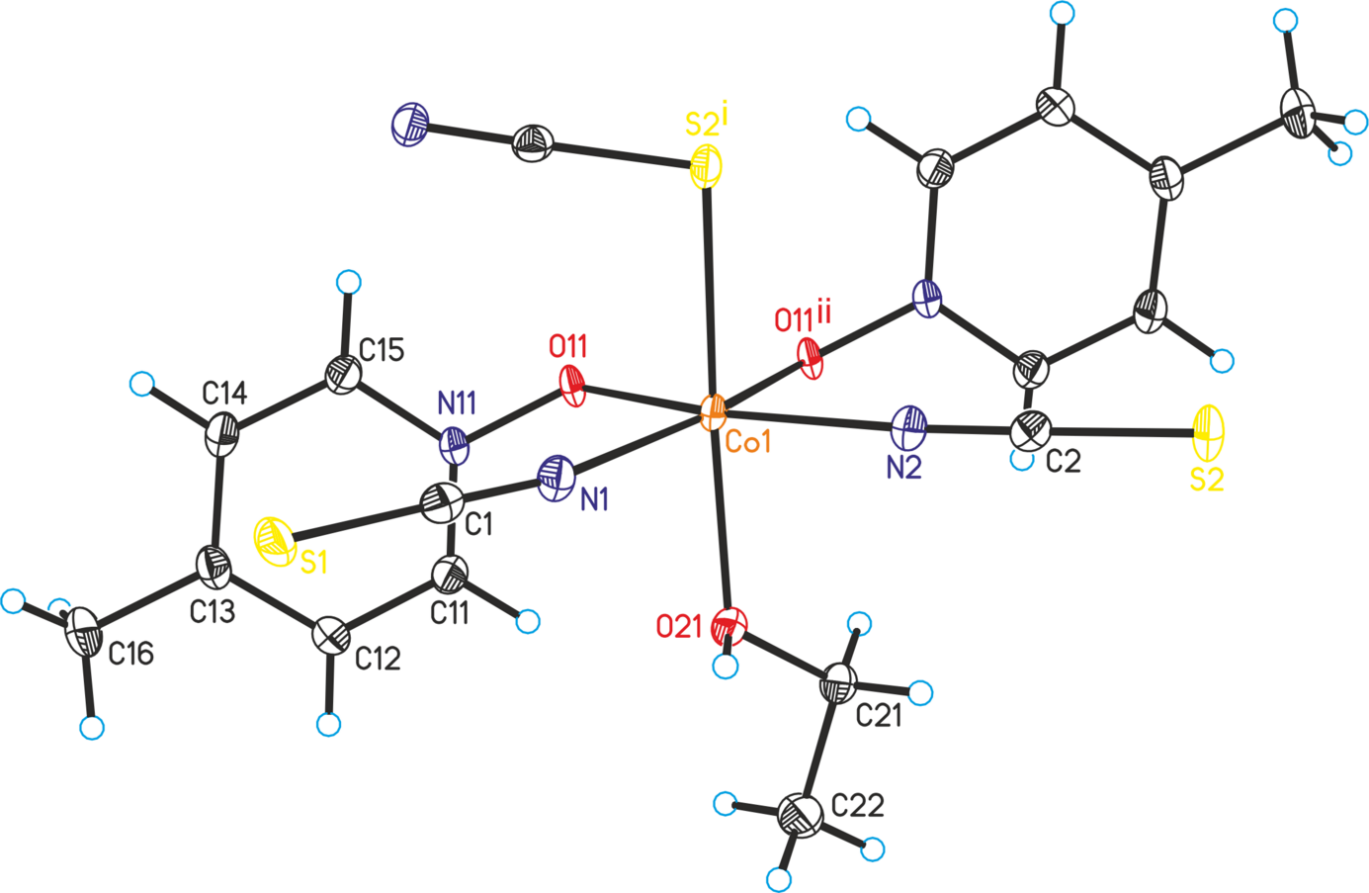
Crystal structure of the title compound with labeling and displacement ellipsoids drawn at the 50% probability level. Symmetry codes: (i) *x*, −*y* + 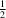, *z* − 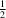; (ii) −*x* + 1, −*y* + 1, −*z* + 1.

**Figure 2:**
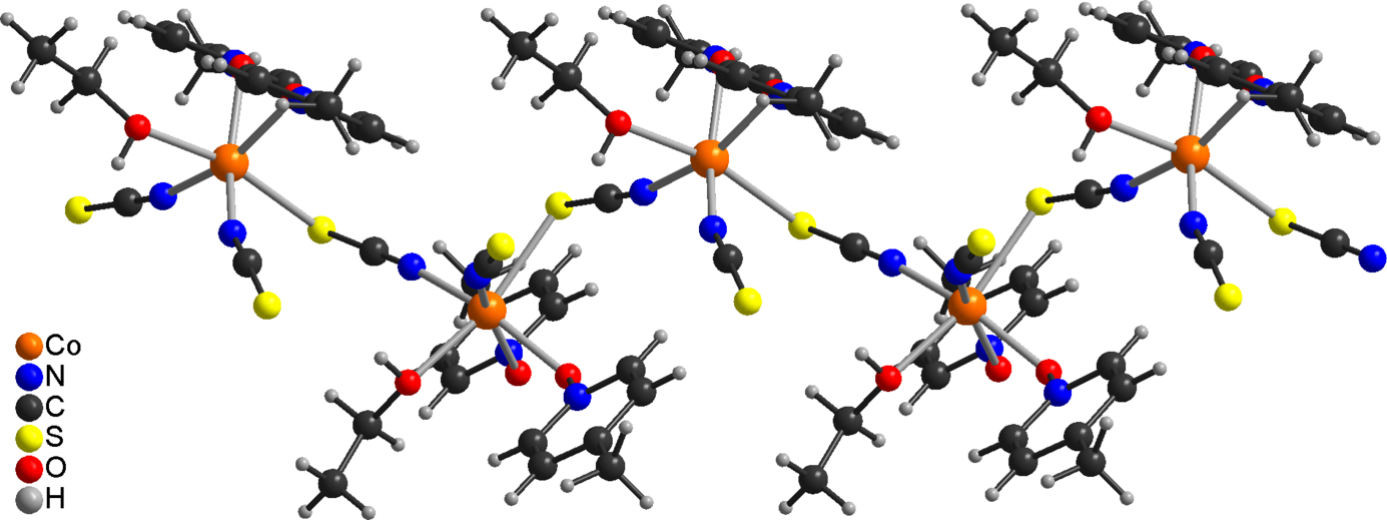
Crystal structure of the title compound with view of a Co(NCS)_2_ chain.

**Figure 3:**
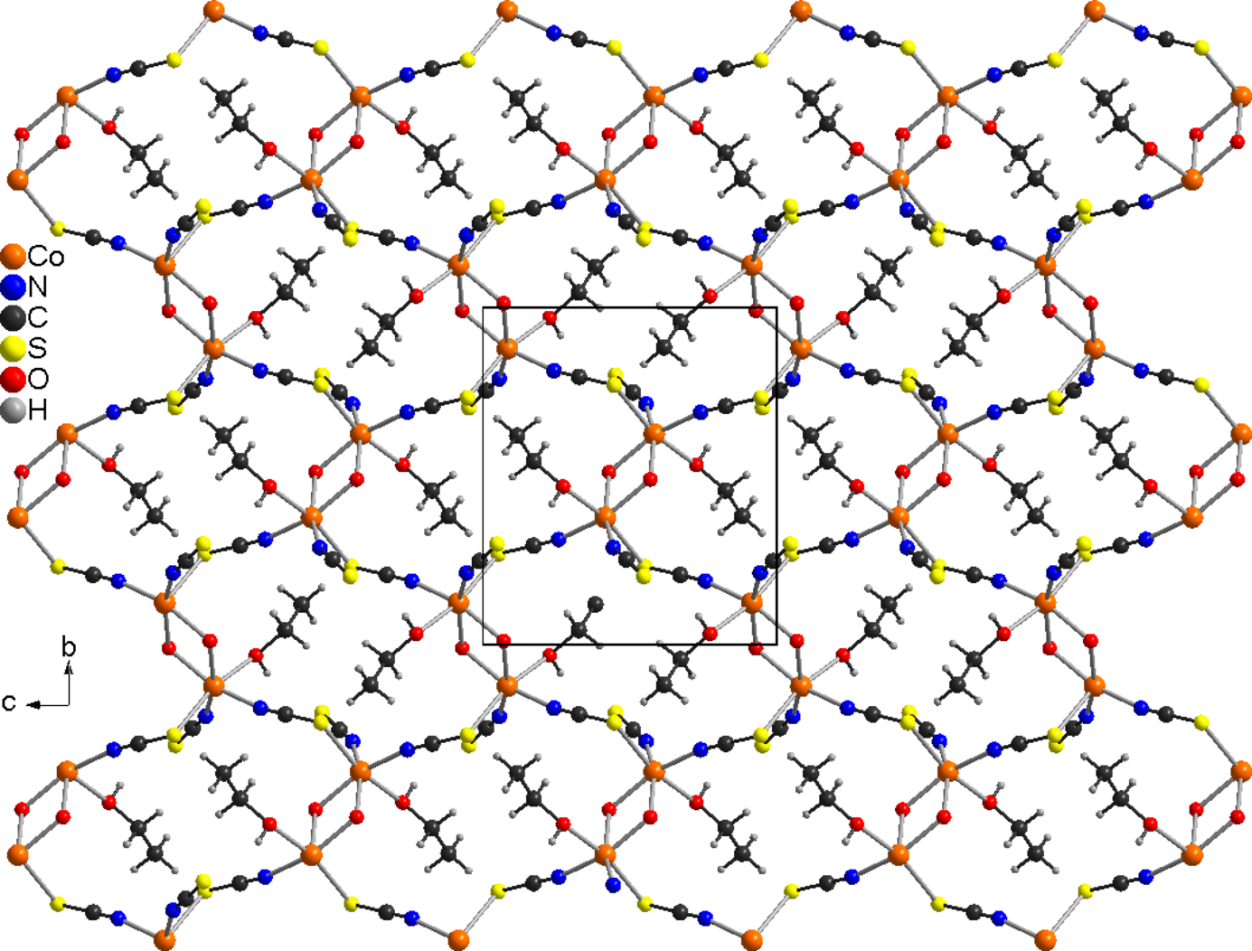
Crystal structure of the title compound with view along the crystallographic *a*-axis, showing the layered network. For the 4-methyl­pyridine *N*-oxide coligands, only the O atoms are shown.

The asymmetric unit of the title compound, Co(NCS)_2_(4-methyl­pyridine *N*-oxide)(ethanol), is built up of one cobalt cation, two crystallographically independent thio­cyanate anions, one ethanol and one 4-methyl­pyridine *N*-oxide coligand that are located in general positions. The Co cations are sixfold coordinated by one terminal N-bonded and two μ-1,3(*N*,*S*)-bridging thio­cyanate anions, one ethanol mol­ecule and two μ-1,1(*O*,*O*)-bridging 4-methyl­pyridine *N*-oxide co­ligands (Fig. 1 ▸). The bridging thio­cyanate anions and the 4-methyl­pyridine *N*-oxide coligands are each in *cis*-positions. The Co—N bond length to the terminal anions is slightly shorter than that to the bridging anionic ligands (Table 1 ▸). The bond angles deviate from ideal values, which shows that a distorted octa­hedral coordination is present (Table 1 ▸). The Co^II^ cations are linked by single μ-1,3(*N*,*S*)-bridging thio­cyanate anions into corrugated chains that proceed along the crystallographic *c*-axis direction (Fig. 2 ▸). The chains are linked by two μ-1,1(*O*,*O*)-bridging 4-methyl­pyridine *N*-oxide co­ligands into layers *via* four-membered Co_2_O_2_ rings (Fig. 3 ▸). These layers consist of large rings built up of six Co^II^ cations, four bridging thio­cyanate anions and two bridging 4-methyl­pyridine *N*-oxide coligands (Fig. 3 ▸). Within these rings, each of the two Co^II^ cations are linked by pairs of μ-1,1(*O*,*O*)-bridg­ing 4-methyl­pyridine *N*-oxide coligands into dinuclear units that are further connected by single μ-1,3-bridging thio­cyanate anions (Fig. 3 ▸).

Even though the overall composition of the title compound is very similar to that of Co(NCS)_2_(4-methyl­pyridine *N*-oxide)(methanol), which has already been reported in the literature, their crystal structures are completely different. In the compound with methanol, the Co^II^ cations are linked by alternating pairs of μ-1,3-bridging thio­cyanate anions and μ-1,1(*O*,*O*)-bridging 4-methyl­pyridine *N*-oxide coligands into chains (Shi *et al.*, 2006*a* ▸). However, layered thio­cyanate networks that also consist of condensed rings are known from compounds with pyridine derivatives as coligands. In, for example, *M*(NCS)_2_(ethyl­isonicotinate)_2_ with *M* = Co, Ni (Suckert *et al.*, 2016 ▸), both metal cations are linked by pairs of μ-1,3-bridging anions into dinuclear units that, as in the title compound, are further connected by single μ-1,3-bridging anionic ligands into layers.

## 3. Supra­molecular features

**Figure 4:**
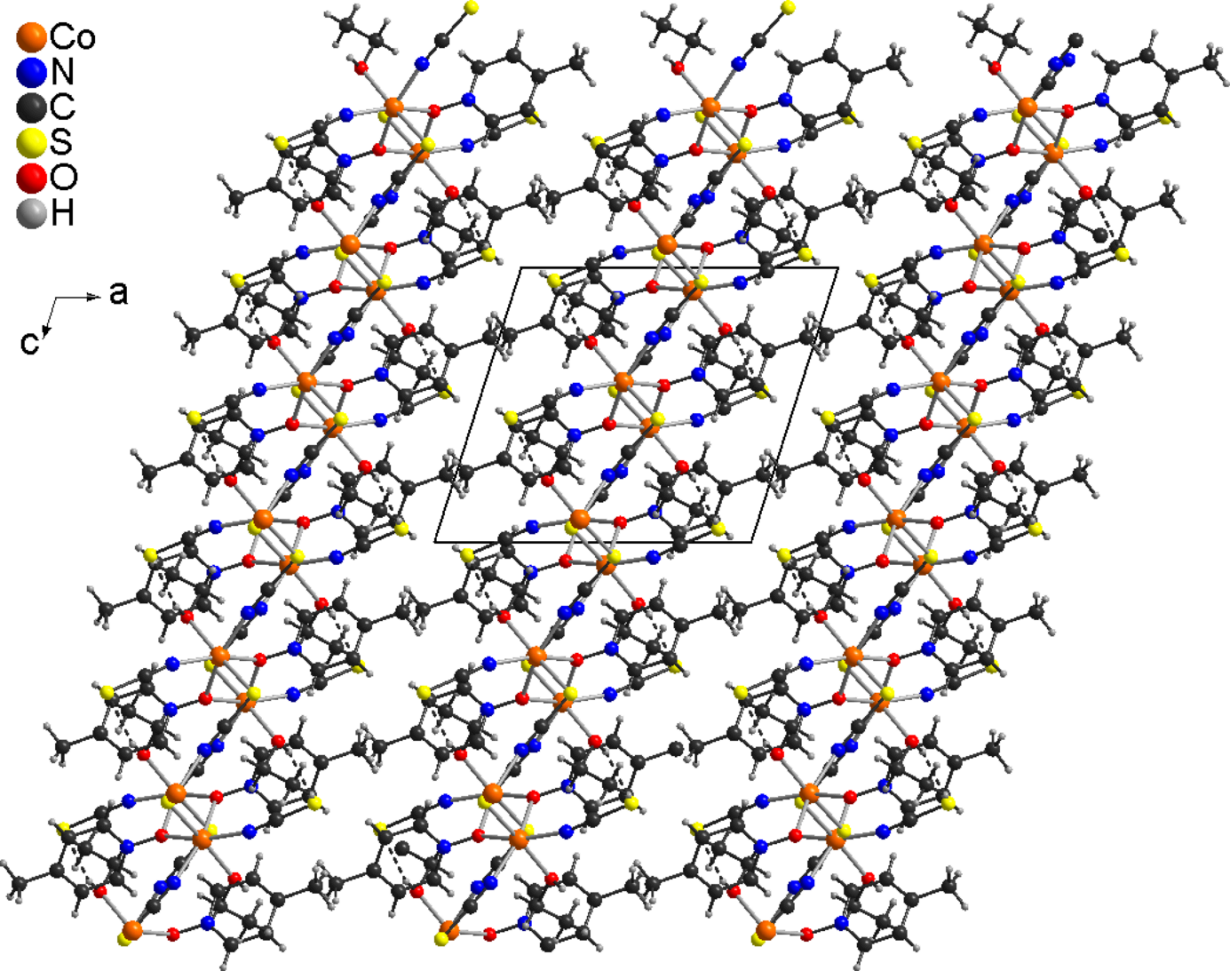
Crystal structure of the title compound with view along the crystallographic *b*-axis, showing the arrangement of the layers. Intra­layer hydrogen bonding is shown as dashed lines.

In the crystal structure of the title compound, the layers are parallel to the *bc* plane and are separated by the methyl groups of the 4-methyl­pyridine *N*-oxide coligands (Fig. 3 ▸). Therefore, no significant inter­molecular inter­actions are observed between the layers (Table 2 ▸). However, intra­layer C—H⋯S and O—H⋯S hydrogen bonding is present with C—H⋯S and O—H⋯S angles close to linearity (Table 2 ▸ and Fig. 4 ▸).

## 4. Database survey

As mentioned above, two compounds based on Co(NCS)_2_ and 4-methyl­pyridine *N*-oxide are already reported in the CSD (version 5.43, last update March 2023; Groom *et al.*, 2016 ▸). These include Co(NCS)_2_(4-methyl­pyridine *N*-oxide) (Ref­code: MEQKOJ, Zhang *et al.*, 2006*a* ▸) and Co(NCS)_2_(4-methyl­pyridine*N*-oxide)(methanol) (Refcode: REKBUF, Shi *et al.*, 2006*a* ▸). Two discrete complexes with the composition Co(NCS)_2_(4-methyl­pyridine *N*-oxide)_3_ and Co(NCS)_2_(4-meth­yl­pyridine *N*-oxide)_4_ (Näther & Jess, 2024*a* ▸) as well as one chain compound with the composition Co_2_(NCS)_4_(4-methyl­pyridine *N*-oxide)_4_(methanol)_2_ (Näther & Jess, 2024*b* ▸) are also reported.

Additionally, several other *M*(NCS)_2_ compounds with 4-methyl­pyridine *N*-oxide are also listed in the CSD. These include *M*(NCS)_2_(4-methyl­pyridine *N*-oxide) with *M* = Ni, Cd) (Refcodes: PEDSUN, Shi *et al.*, 2006*b* ▸; PEDSUN01, Marsh, 2009 ▸; TEQKAC, Shi *et al.*, 2006*c* ▸). With copper(II), a compound with the composition Cu(NCS)_2_(4-methyl­pyridine *N*-oxide) is reported in which the Cu^II^ cations are octa­hedrally coordinated and linked into chains by pairs of bridging thio­cyanate anions, which are further connected into double chains *via* Cu_2_S_2_ rings (Refcode TEBTAW, Shi *et al.*, 2006*d* ▸). With Ni^II^ and Mn^II^, two discrete aqua complexes with the composition *M*(NCS)_2_(4-methyl­pyridine *N*-oxide)_2_(H_2_O)_2_ (*M* = Ni, Shi *et al.*, 2006*a* ▸ and *M* = Mn, Mautner *et al.*, 2018*a* ▸,*b* ▸) are also reported. Three isotypic compounds with the composition *M*(NCS)_2_)(acetato)_2_(H_2_O)_3_(4-methyl­pyridine *N*-oxide) with *M* = Sm, Eu, Gd) are also known (Refcodes: GIHBUV, Zhang & Shi, 2007 ▸; PIJBIU and PIJBOA, Shi *et al.*, 2007*a* ▸).

Some Co(NCS)_2_ compounds with other pyridine *N*-oxide derivatives are also known. These include Co(NCS)_2_(pyridine *N*-oxide)_2_(H_2_O)_2_ and Co(NCS)_2_(3-hy­droxy­pyridine *N*-oxide)_2_(H_2_O)_2_, which consist of discrete octa­hedral complexes (Refcodes: FONBIU, Shi *et al.*, 2005 ▸; IDOYEG, Shi *et al.*, 2006*e* ▸). They also include Co(NCS)_2_(4-meth­oxy­pyridine *N*-oxide), which is isotypic to its 4-methyl­pyridine analog (Refcode TERRAK, Zhang *et al.*, 2006*b* ▸) and Co(NCS)_2_(4-nitro­pyridine *N*-oxide) (Shi *et al.*, 2007*b* ▸). Finally, we have also reported some Co(NCS)_2_ compounds with pyridine *N*-oxide derivatives, including Co(NCS)_2_(3-cyano­pyridine *N*-oxide)_4_ (Näther & Jess, 2023 ▸), Co(NCS)_2_(2-methyl­pyridine *N*-oxide) (Näther & Jess, 2024*c* ▸), and Co(NCS)_2_(2-methyl­pyridine *N*-oxide)_3_ (Näther & Jess, 2024*d* ▸).

## 5. Synthesis and crystallization

Co(NCS)_2_ (99%) was purchased from Sigma Aldrich and 4-methyl­pyridine *N*-oxide (97%) from Thermo Scientific.

**Synthesis:**

Crystals of the title compound were accidentally obtained by the reaction of 0.5 mmol (87 mg) Co(SCN)_2_ and 0.5 mmol (54 mg) of 4-methyl­pyridine *N*-oxide in 1 mL of ethanol. The reaction mixture was stored overnight, which led to the formation of a violet-colored crystalline precipitate. X-ray powder diffraction measurements prove that the majority of the sample consists of the known discrete complex Co(NCS)_2_(4-methyl­pyridine *N*-oxide)_3_ (Näther & Jess, 2024*a* ▸) and that only traces of the title compound are present.

## 6. Refinement

Crystal data, data collection and structure refinement details are summarized in Table 3 ▸. The C—H hydrogen atoms were positioned with idealized geometry (methyl H atoms allowed to rotate and not to tip) and were refined with *U*_iso_(H) = 1.2*U*_eq_(C) (1.5 for methyl H atoms) using a riding model.

## Supplementary Material

Crystal structure: contains datablock(s) I. DOI: 10.1107/S2056989024009058/pk2710sup1.cif

Structure factors: contains datablock(s) I. DOI: 10.1107/S2056989024009058/pk2710Isup2.hkl

CCDC reference: 2384436

Additional supporting information:  crystallographic information; 3D view; checkCIF report

## Figures and Tables

**Table 1:** Selected geometric parameters (Å, °)

Co1—N1	2.0472 (13)	Co1—O11	2.0971 (10)
Co1—N2	2.0569 (12)	Co1—O11^ii^	2.1594 (10)
Co1—S2^i^	2.5171 (4)	Co1—O21	2.1559 (10)
			
N1—Co1—N2	110.80 (5)	N2—Co1—O21	86.53 (5)
N1—Co1—S2^i^	90.75 (4)	O11—Co1—S2^i^	98.02 (3)
N1—Co1—O11^ii^	160.49 (5)	O11^ii^—Co1—S2^i^	95.96 (3)
N1—Co1—O11	89.92 (4)	O11—Co1—O11^ii^	71.03 (4)
N1—Co1—O21	82.55 (5)	O11—Co1—O21	92.89 (4)
N2—Co1—S2^i^	85.67 (4)	O21—Co1—S2^i^	167.20 (3)
N2—Co1—O11^ii^	88.01 (4)	O21—Co1—O11^ii^	93.90 (4)
N2—Co1—O11	158.96 (5)		

Symmetry codes: (i) 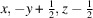; (ii) 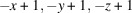.

**Table 2:** Hydrogen-bond geometry (Å, °)

*D*—H⋯*A*	*D*—H	H⋯*A*	*D*⋯*A*	*D*—H⋯*A*
C11—H11⋯S2^iii^	0.95	2.95	3.4900 (15)	118
C11—H11⋯O21	0.95	2.57	3.2004 (18)	124
C12—H12⋯S1^iv^	0.95	2.96	3.8544 (16)	157
O21—H21⋯S1^v^	0.82 (2)	2.53 (2)	3.3028 (11)	159 (2)
C22—H22*C*⋯S2^vi^	0.98	2.93	3.7185 (18)	138

Symmetry codes: (iii) 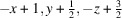; (iv) 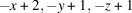; (v) 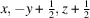; (vi) 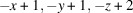.

**Table 3:** Experimental details

Crystal data	
Chemical formula	[Co(NCS)_2_(C_6_H_7_NO)(C_2_H_6_O)]
*M*_r_	330.28
Crystal system, space group	Monoclinic, *P*2_1_/*c*
Temperature (K)	100
*a*, *b*, *c* (Å)	11.67627 (7), 11.61861 (6), 10.60662 (7)
β (°)	107.5929 (7)
*V* (Å^3^)	1371.62 (2)
*Z*	4
Radiation type	Cu *K*α
μ (mm^−1^)	12.65
Crystal size (mm)	0.15 × 0.12 × 0.10 × 0.08 (radius)

Data collection	
Diffractometer	XtaLAB Synergy, Dualflex, HyPix
Absorption correction	Multi-scan (*CrysAlis PRO*; Rigaku OD, 2023 ▸)
*T*_min_, *T*_max_	0.453, 1.000
No. of measured, independent and observed [*I* > 2σ(*I*)] reflections	23514, 2937, 2909
*R*_int_	0.022
(sin θ/λ)_max_ (Å^−1^)	0.639

Refinement	
*R*[*F*^2^ > 2σ(*F*^2^)], *wR*(*F*^2^), *S*	0.022, 0.058, 1.05
No. of reflections	2937
No. of parameters	170
No. of restraints	1
H-atom treatment	H atoms treated by a mixture of independent and constrained refinement
Δρ_max_, Δρ_min_ (e Å^−3^)	0.35, −0.29

Computer programs: *CrysAlis PRO* (Rigaku OD, 2023 ▸), *SHELXT2014/5* (Sheldrick, 2015*a* ▸), *SHELXL2016/6* (Sheldrick, 2015*b* ▸), *DIAMOND* (Brandenburg, 1999 ▸) and *XP* in *SHELXTL-PC* (Sheldrick, 2008 ▸) and *publCIF* (Westrip, 2010 ▸).

## supplementary crystallographic information

### Poly[ethanol(µ-4-methylpyridine N-oxide)di-µ-thiocyanato-cobalt(II)] Crystal data

**Table:**

[Co(NCS)_2_(C_6_H_7_NO)(C_2_H_6_O)]	*F*(000) = 676
*M**_r_* = 330.28	*D*_x_ = 1.599 Mg m^−^^3^
Monoclinic, *P*2_1_/*c*	Cu *K*α radiation, λ = 1.54184 Å
*a* = 11.67627 (7) Å	Cell parameters from 19460 reflections
*b* = 11.61861 (6) Å	θ = 4.0–79.9°
*c* = 10.60662 (7) Å	µ = 12.65 mm^−^^1^
β = 107.5929 (7)°	*T* = 100 K
*V* = 1371.62 (2) Å^3^	Block, pink
*Z* = 4	0.15 × 0.12 × 0.10 × 0.08 (radius) mm

### Poly[ethanol(µ-4-methylpyridine N-oxide)di-µ-thiocyanato-cobalt(II)] Data collection

**Table:**

XtaLAB Synergy, Dualflex, HyPix diffractometer	2937 independent reflections
Radiation source: micro-focus sealed X-ray tube, PhotonJet (Cu) X-ray Source	2909 reflections with *I* > 2σ(*I*)
Mirror monochromator	*R*_int_ = 0.022
Detector resolution: 10.0000 pixels mm^-1^	θ_max_ = 80.4°, θ_min_ = 4.0°
ω scans	*h* = −14→13
Absorption correction: multi-scan (CrysAlisPro; Rigaku OD, 2023)	*k* = −14→14
*T*_min_ = 0.453, *T*_max_ = 1.000	*l* = −11→13
23514 measured reflections	

### Poly[ethanol(µ-4-methylpyridine N-oxide)di-µ-thiocyanato-cobalt(II)] Refinement

**Table:**

Refinement on *F*^2^	Hydrogen site location: mixed
Least-squares matrix: full	H atoms treated by a mixture of independent and constrained refinement
*R*[*F*^2^ > 2σ(*F*^2^)] = 0.022	*w* = 1/[σ^2^(*F*_o_^2^) + (0.0303*P*)^2^ + 0.9886*P*] where *P* = (*F*_o_^2^ + 2*F*_c_^2^)/3
*wR*(*F*^2^) = 0.058	(Δ/σ)_max_ = 0.001
*S* = 1.05	Δρ_max_ = 0.35 e Å^−^^3^
2937 reflections	Δρ_min_ = −0.29 e Å^−^^3^
170 parameters	Extinction correction: *SHELXL2016/6* (Sheldrick, 2015b), Fc^*^=kFc[1+0.001xFc^2^λ^3^/sin(2θ)]^-1/4^
1 restraint	Extinction coefficient: 0.00084 (11)
Primary atom site location: dual	

### Poly[ethanol(µ-4-methylpyridine N-oxide)di-µ-thiocyanato-cobalt(II)] Special details

**Table:**

Geometry. All esds (except the esd in the dihedral angle between two l.s. planes) are estimated using the full covariance matrix. The cell esds are taken into account individually in the estimation of esds in distances, angles and torsion angles; correlations between esds in cell parameters are only used when they are defined by crystal symmetry. An approximate (isotropic) treatment of cell esds is used for estimating esds involving l.s. planes.

### Poly[ethanol(µ-4-methylpyridine N-oxide)di-µ-thiocyanato-cobalt(II)] Fractional atomic coordinates and isotropic or equivalent isotropic displacement parameters (Å^2^)

**Table:**

	*x*	*y*	*z*	*U*_iso_*/*U*_eq_	
Co1	0.56402 (2)	0.37657 (2)	0.58362 (2)	0.01116 (8)	
N1	0.70562 (11)	0.28747 (11)	0.55557 (12)	0.0163 (3)	
C1	0.77833 (13)	0.25258 (12)	0.51159 (14)	0.0156 (3)	
S1	0.88296 (3)	0.20182 (3)	0.45220 (4)	0.02192 (10)	
N2	0.51802 (11)	0.31162 (11)	0.74214 (12)	0.0155 (2)	
C2	0.47782 (13)	0.29336 (12)	0.82748 (14)	0.0143 (3)	
S2	0.41839 (3)	0.26862 (3)	0.94760 (3)	0.01787 (9)	
O11	0.56699 (9)	0.48910 (9)	0.43004 (10)	0.0133 (2)	
N11	0.66546 (11)	0.50302 (10)	0.38914 (12)	0.0123 (2)	
C11	0.76069 (13)	0.56068 (13)	0.46681 (14)	0.0164 (3)	
H11	0.759449	0.589098	0.550371	0.020*	
C12	0.86013 (13)	0.57840 (13)	0.42474 (15)	0.0179 (3)	
H12	0.927076	0.619664	0.479306	0.021*	
C13	0.86310 (13)	0.53621 (13)	0.30276 (15)	0.0175 (3)	
C14	0.76325 (14)	0.47485 (13)	0.22755 (15)	0.0182 (3)	
H14	0.762990	0.443743	0.144637	0.022*	
C15	0.66507 (13)	0.45867 (13)	0.27160 (14)	0.0164 (3)	
H15	0.597523	0.416577	0.219592	0.020*	
C16	0.96938 (15)	0.55720 (18)	0.25409 (17)	0.0289 (4)	
H16A	0.984966	0.488350	0.208476	0.043*	
H16B	1.040050	0.574519	0.329344	0.043*	
H16C	0.952495	0.622453	0.192663	0.043*	
O21	0.70560 (9)	0.46801 (9)	0.72670 (10)	0.0153 (2)	
H21	0.7507 (19)	0.4167 (18)	0.764 (2)	0.036 (6)*	
C21	0.68639 (13)	0.54339 (13)	0.82663 (15)	0.0173 (3)	
H21A	0.671358	0.496901	0.898243	0.021*	
H21B	0.614811	0.591950	0.787326	0.021*	
C22	0.79487 (16)	0.61910 (14)	0.88314 (18)	0.0248 (4)	
H22A	0.808629	0.666175	0.812428	0.037*	
H22B	0.865522	0.570888	0.922494	0.037*	
H22C	0.780882	0.669390	0.951198	0.037*	

### Poly[ethanol(µ-4-methylpyridine N-oxide)di-µ-thiocyanato-cobalt(II)] Atomic displacement parameters (Å^2^)

**Table:**

	*U*^11^	*U*^22^	*U*^33^	*U*^12^	*U*^13^	*U*^23^
Co1	0.01275 (12)	0.01291 (13)	0.00940 (12)	0.00043 (8)	0.00570 (9)	0.00058 (8)
N1	0.0177 (6)	0.0179 (6)	0.0145 (6)	0.0026 (5)	0.0067 (5)	0.0004 (5)
C1	0.0164 (7)	0.0152 (7)	0.0144 (6)	−0.0010 (5)	0.0033 (5)	−0.0004 (5)
S1	0.01501 (17)	0.0262 (2)	0.0272 (2)	−0.00084 (14)	0.01044 (14)	−0.00874 (15)
N2	0.0178 (6)	0.0167 (6)	0.0136 (6)	0.0006 (5)	0.0070 (5)	0.0013 (5)
C2	0.0151 (6)	0.0137 (6)	0.0135 (6)	0.0023 (5)	0.0033 (5)	0.0004 (5)
S2	0.02135 (18)	0.02055 (18)	0.01625 (17)	0.00594 (13)	0.01250 (14)	0.00563 (13)
O11	0.0125 (5)	0.0164 (5)	0.0145 (5)	0.0014 (4)	0.0092 (4)	0.0033 (4)
N11	0.0127 (5)	0.0132 (5)	0.0130 (5)	0.0007 (4)	0.0067 (4)	0.0018 (4)
C11	0.0170 (7)	0.0187 (7)	0.0139 (6)	0.0007 (6)	0.0051 (5)	−0.0004 (5)
C12	0.0149 (7)	0.0213 (7)	0.0181 (7)	−0.0022 (6)	0.0060 (5)	−0.0009 (6)
C13	0.0162 (7)	0.0206 (7)	0.0181 (7)	0.0010 (6)	0.0087 (6)	0.0031 (6)
C14	0.0206 (7)	0.0212 (7)	0.0152 (7)	0.0013 (6)	0.0092 (6)	−0.0008 (6)
C15	0.0183 (7)	0.0173 (7)	0.0145 (6)	−0.0017 (6)	0.0063 (5)	−0.0021 (5)
C16	0.0216 (8)	0.0459 (11)	0.0242 (8)	−0.0054 (7)	0.0143 (7)	−0.0012 (7)
O21	0.0154 (5)	0.0180 (5)	0.0130 (5)	0.0007 (4)	0.0052 (4)	−0.0017 (4)
C21	0.0169 (7)	0.0196 (7)	0.0165 (7)	0.0011 (6)	0.0067 (6)	−0.0039 (6)
C22	0.0225 (8)	0.0266 (9)	0.0263 (8)	−0.0042 (6)	0.0088 (7)	−0.0098 (6)

### Poly[ethanol(µ-4-methylpyridine N-oxide)di-µ-thiocyanato-cobalt(II)] Geometric parameters (Å, º)

**Table:**

Co1—N1	2.0472 (13)	C13—C14	1.393 (2)
Co1—N2	2.0569 (12)	C13—C16	1.501 (2)
Co1—S2^i^	2.5171 (4)	C14—H14	0.9500
Co1—O11	2.0971 (10)	C14—C15	1.375 (2)
Co1—O11^ii^	2.1594 (10)	C15—H15	0.9500
Co1—O21	2.1559 (10)	C16—H16A	0.9800
N1—C1	1.158 (2)	C16—H16B	0.9800
C1—S1	1.6438 (15)	C16—H16C	0.9800
N2—C2	1.158 (2)	O21—H21	0.815 (16)
C2—S2	1.6498 (15)	O21—C21	1.4437 (17)
O11—N11	1.3557 (15)	C21—H21A	0.9900
N11—C11	1.3454 (19)	C21—H21B	0.9900
N11—C15	1.3477 (18)	C21—C22	1.509 (2)
C11—H11	0.9500	C22—H22A	0.9800
C11—C12	1.379 (2)	C22—H22B	0.9800
C12—H12	0.9500	C22—H22C	0.9800
C12—C13	1.394 (2)		
			
N1—Co1—N2	110.80 (5)	C13—C12—H12	119.8
N1—Co1—S2^i^	90.75 (4)	C12—C13—C16	121.15 (14)
N1—Co1—O11^ii^	160.49 (5)	C14—C13—C12	117.39 (14)
N1—Co1—O11	89.92 (4)	C14—C13—C16	121.46 (14)
N1—Co1—O21	82.55 (5)	C13—C14—H14	119.5
N2—Co1—S2^i^	85.67 (4)	C15—C14—C13	120.97 (14)
N2—Co1—O11^ii^	88.01 (4)	C15—C14—H14	119.5
N2—Co1—O11	158.96 (5)	N11—C15—C14	119.46 (14)
N2—Co1—O21	86.53 (5)	N11—C15—H15	120.3
O11—Co1—S2^i^	98.02 (3)	C14—C15—H15	120.3
O11^ii^—Co1—S2^i^	95.96 (3)	C13—C16—H16A	109.5
O11—Co1—O11^ii^	71.03 (4)	C13—C16—H16B	109.5
O11—Co1—O21	92.89 (4)	C13—C16—H16C	109.5
O21—Co1—S2^i^	167.20 (3)	H16A—C16—H16B	109.5
O21—Co1—O11^ii^	93.90 (4)	H16A—C16—H16C	109.5
C1—N1—Co1	163.29 (12)	H16B—C16—H16C	109.5
N1—C1—S1	178.79 (14)	Co1—O21—H21	103.2 (17)
C2—N2—Co1	166.81 (12)	C21—O21—Co1	124.02 (9)
N2—C2—S2	178.99 (14)	C21—O21—H21	107.9 (17)
C2—S2—Co1^iii^	100.98 (5)	O21—C21—H21A	109.6
Co1—O11—Co1^ii^	108.96 (4)	O21—C21—H21B	109.6
N11—O11—Co1	122.53 (8)	O21—C21—C22	110.31 (12)
N11—O11—Co1^ii^	122.99 (8)	H21A—C21—H21B	108.1
C11—N11—O11	119.16 (12)	C22—C21—H21A	109.6
C11—N11—C15	121.87 (12)	C22—C21—H21B	109.6
C15—N11—O11	118.97 (12)	C21—C22—H22A	109.5
N11—C11—H11	120.1	C21—C22—H22B	109.5
N11—C11—C12	119.79 (13)	C21—C22—H22C	109.5
C12—C11—H11	120.1	H22A—C22—H22B	109.5
C11—C12—H12	119.8	H22A—C22—H22C	109.5
C11—C12—C13	120.49 (14)	H22B—C22—H22C	109.5

Symmetry codes: (i) *x*, −*y*+1/2, *z*−1/2; (ii) −*x*+1, −*y*+1, −*z*+1; (iii) *x*, −*y*+1/2, *z*+1/2.

### Poly[ethanol(µ-4-methylpyridine N-oxide)di-µ-thiocyanato-cobalt(II)] Hydrogen-bond geometry (Å, º)

**Table:**

*D*—H···*A*	*D*—H	H···*A*	*D*···*A*	*D*—H···*A*
C11—H11···S2^iv^	0.95	2.95	3.4900 (15)	118
C11—H11···O21	0.95	2.57	3.2004 (18)	124
C12—H12···S1^v^	0.95	2.96	3.8544 (16)	157
O21—H21···S1^iii^	0.82 (2)	2.53 (2)	3.3028 (11)	159 (2)
C22—H22*C*···S2^vi^	0.98	2.93	3.7185 (18)	138

Symmetry codes: (iii) *x*, −*y*+1/2, *z*+1/2; (iv) −*x*+1, *y*+1/2, −*z*+3/2; (v) −*x*+2, −*y*+1, −*z*+1; (vi) −*x*+1, −*y*+1, −*z*+2.
